# Molecular identification of an immunity- and Ferroptosis-related gene signature in non-small cell lung Cancer

**DOI:** 10.1186/s12885-021-08541-w

**Published:** 2021-07-06

**Authors:** Taisheng Liu, Honglian Luo, Jinye Zhang, Xiaoshan Hu, Jian Zhang

**Affiliations:** 1 Department of Thoracic Surgery, Affiliated Cancer Hospital & Institute of Guangzhou Medical University, Guangzhou, 510095 P. R. China; 2 College of Oncology, Guangxi Medical University, Nanning, 530021 P. R. China; 3 Internal Medicine of Oncology, Affiliated Cancer Hospital & Institute of Guangzhou Medical University, Guangzhou, 510095 P. R. China; 4 Department of Radiation Oncology, State Key Laboratory of Respiratory Diseases, Affiliated Cancer Hospital & Institute of Guangzhou Medical University, Guangzhou Institute of Respiratory Disease, Guangzhou, 510095 P. R. China

**Keywords:** Lung cancer, Ferroptosis, Immunity, Overall survival, Gene signature

## Abstract

**Background:**

Lung cancer is one of the dominant causes of cancer-related deaths worldwide. Ferroptosis, an iron-dependent form of programmed cell death, plays a key role in cancer immunotherapy. However, the role of immunity- and ferroptosis-related gene signatures in non-small cell lung cancer (NSCLC) remain unclear.

**Methods:**

RNA-seq data and clinical information pertaining to NSCLC were collected from The Cancer Genome Atlas dataset. Univariate and multivariate Cox regression analyses were performed to identify ferroptosis-related genes. A receiver operating characteristic (ROC) model was established for sensitivity and specificity evaluation. Gene ontology enrichment and Kyoto Encyclopedia of Genes and Genomes pathway analyses were performed to explore the function roles of differentially expressed genes.

**Results:**

A signature composed of five ferroptosis-related genes was established to stratify patients into high- and low-risk subgroups. In comparison with patients in the low-risk group, those in the high-risk one showed significantly poor overall survival in the training and validation cohorts (*P* < 0.05). Multivariate Cox regression analysis indicated risk score to be an independent predictor of overall survival (*P* < 0.01). Further, the 1-, 2-, and 3-year ROCs were 0.623 vs. 0.792 vs. 0.635, 0.644 vs. 0.792 vs. 0.634, and 0.631 vs. 0.641 vs. 0.666 in one training and two validation cohorts, respectively. Functional analysis revealed that immune-related pathways were enriched and associated with abnormal activation of immune cells.

**Conclusions:**

We identified five immunity- and ferroptosis-related genes that may be involved in NSCLC progression and prognosis. Targeting ferroptosis-related genes seems to be an alternative to clinical therapy for NSCLC.

**Supplementary Information:**

The online version contains supplementary material available at 10.1186/s12885-021-08541-w.

## Background

Lung cancer has become the leading deadly malignancy across the globe [1], with non-small cell lung cancer (NSCLC) accounting for > 85% of all cases [2]. Despite extensive research on molecular targeted therapies and checkpoint inhibitors, > 50% patients die within 1 year of NSCLC diagnosis, and the 5-year overall survival (OS) rate is < 18% [3]. These data indicate that there is an urgent need for not only novel therapeutic research but also comprehensive analyses to elucidate the molecular mechanisms underlying NSCLC, which should facilitate the identification of new therapeutic targets.

Ferroptosis, an iron-dependent form of programmed cell death, chiefly relies on iron accumulation [4–6]. Emerging evidence shows that ferroptosis is closely related to the development of several human diseases, particularly cancer [7–11]. Ferroptosis has been identified to be a novel way to induce cancer cell death [12–14]. Moreover, ferroptotic cancer cells evidently produce a plethora of oxidized lipid mediators to affect anti-tumor immunity, and a small proportion of cells undergoing ferroptosis are capable of suppressing the immune system, enhancing tumor growth [15]. The induction of ferroptosis can also affect the anti-tumor efficacy of immunotherapy, suggesting that the immune system, at least in part, functions via ferroptosis [16]. However, the relationship between NSCLC patient prognosis and immunity- and ferroptosis-related genes remains unknown, making the development of ferroptosis therapy for NSCLC a major challenge.

In this study, we collected and analyzed of ferroptosis-related NSCLC from The Cancer Genome Atlas (TCGA) dataset and Gene Expression Omnibus (GEO) database. Five immunity- and ferroptosis-related differentially expressed genes (DEGs) were identified to establish a risk model. Patients with NSCLC in the GEO database were selected as the validation cohort. Gene ontology (GO) enrichment and Kyoto Encyclopedia of Genes and Genomes (KEGG) pathway analyses were performed to explore the functions and pathways enriched between the high- and low-risk subgroups. Furthermore, to assess prognosis, a nomogram model was developed based on risk score and clinical features. We believe that our immunity- and ferroptosis-related risk model can serve as a potential gene signature and therapeutic target for NSCLC.

## Methods

### Data acquisition

RNA-seq data (*n* = 594) and clinical information related to NSCLC were obtained from TCGA dataset (https://tcga-data.nci.nih.gov/tcga/). To validate the findings in TCGA dataset, the independent cohorts GSE13213 (*n* = 117) and GSE72904 (*n* = 442) from the GEO database (https://www.ncbi.nlm.nih.gov/geo/) were employed. RNA-seq data and clinical information pertaining to three datasets were independently reviewed by two authors (TX-L and JY-Z) to avoid potential mistakes.

### DEG identification

Sixty ferroptosis-related genes were retrieved from previous literature [14, 17–19]; (Table S1). Sixteen ferroptosis-related DEGs between tumor and normal tissues were identified (Table S1), and 14 ferroptosis-related prognostic genes were found in TCGA database by excluding normal samples (*n* = 59) and tumor samples without or with unknown follow-up information (*n* = 26) using the “limma” R package (version 3.6.2, https://cran.r-project.org/) with the Wilcoxon test. The cut-off criteria were false discovery rate < 0.05 and |log_2_FoldChange| > 1. Univariate and multivariate Cox regressions were used to evaluate the relationship between DEGs and OS. Patients were stratified into high- and low-risk subgroups according to their risk score, which was calculated as follows: risk score = ∑^j^_(*n* = 1)_Coef_j_*X_j_, wherein Coef_j_ represents the coefficient and X_j_ represents the relative expression level of each DEG standardized by z-score.

### Development of receiver operating characteristic (ROC) curves

Univariate Cox regression was used to analyze prognostic DEGs with clinical information. Significant prognostic DEGs (*P* < 0.05) were then analyzed via multivariate Cox regression to identify independent prognostic risk factors. ROC analysis was performed to determine the sensitivity and specificity of the risk model in predicting OS.

### Principal components analysis (PCA) and t-distributed stochastic neighbor embedding (t-SNE)

PCA and t-SNE were used for dimensionality reduction analysis. Based on expression levels of the genes in the signature, PCA was performed using the “prcomp” function of the “stats” R package. In addition, t-SNE was performed using the “Rtsne” package to explore the distribution of different subgroups.

### Interaction network and enrichment analysis

An interaction network of DEGs was constructed using STRING (http://string-db.org/cgi/input.pl). GO enrichment and KEGG pathway analyses were performed to analyze the functions of differential expressed immune-related genes using the R software.

### Immune cells and Ferroptosis

The infiltrating score of 16 types of immune cells and 13 immune-related functions were calculated via single-sample gene set enrichment analysis (ssGSEA) in the “gsva” R package.

### Statistical analysis

Statistical analysis was performed using R v3.6.2 (https://cran.r-project.org/). Student’s *t*-test was used to evaluate differences between groups. ssGSEA scores between the high- and low-risk subgroups were compared with the Mann–Whitney test. The Kaplan–Meier method was used to assess OS and differences were assessed using two-sided log-rank test. Two-sided *P* < 0.05 indicated a statistically significant difference.

## Results

### Identification of prognostic Ferroptosis-related DEGs

**Fig. 1:**
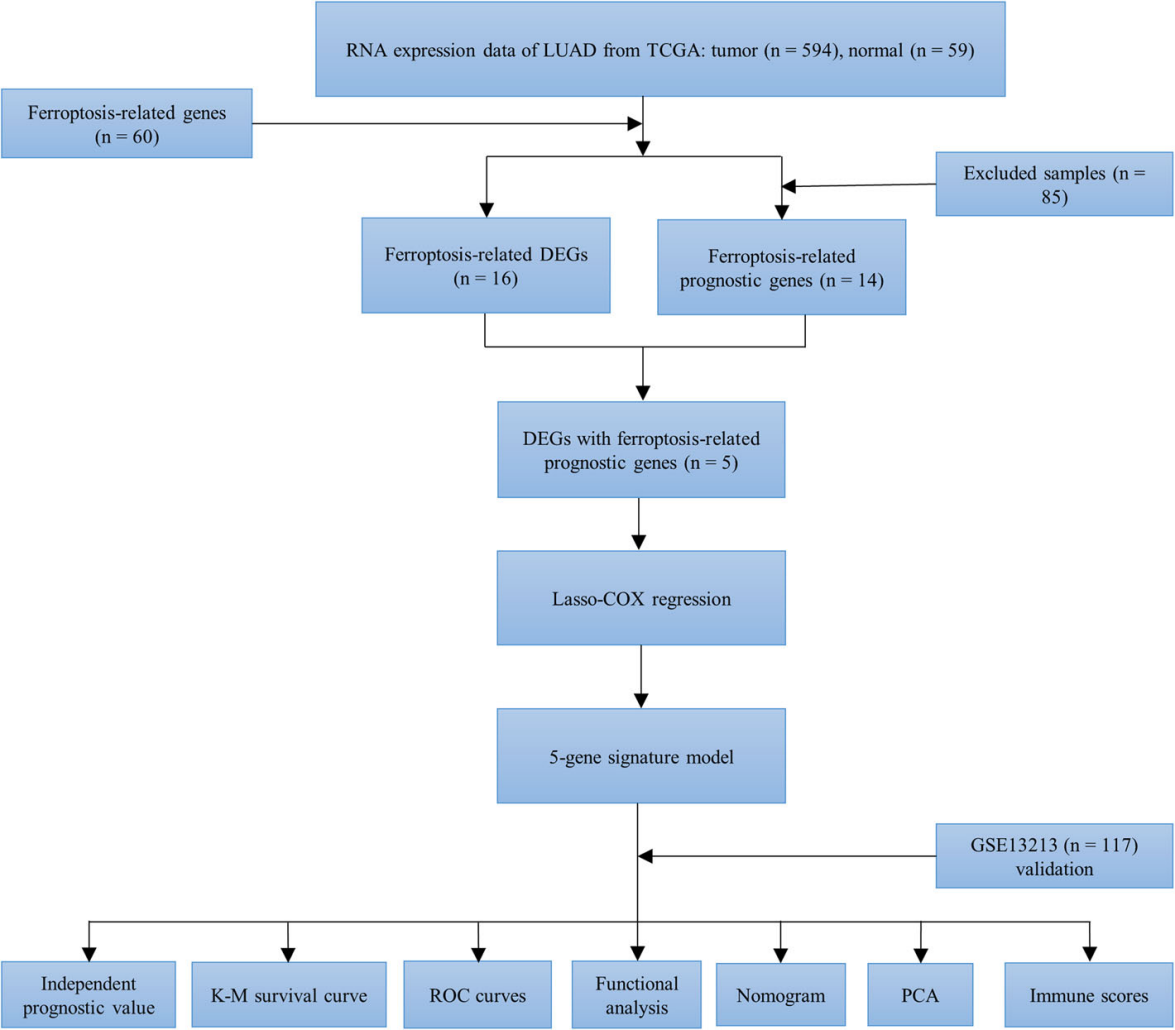
Work flow chart

**Fig. 2:**
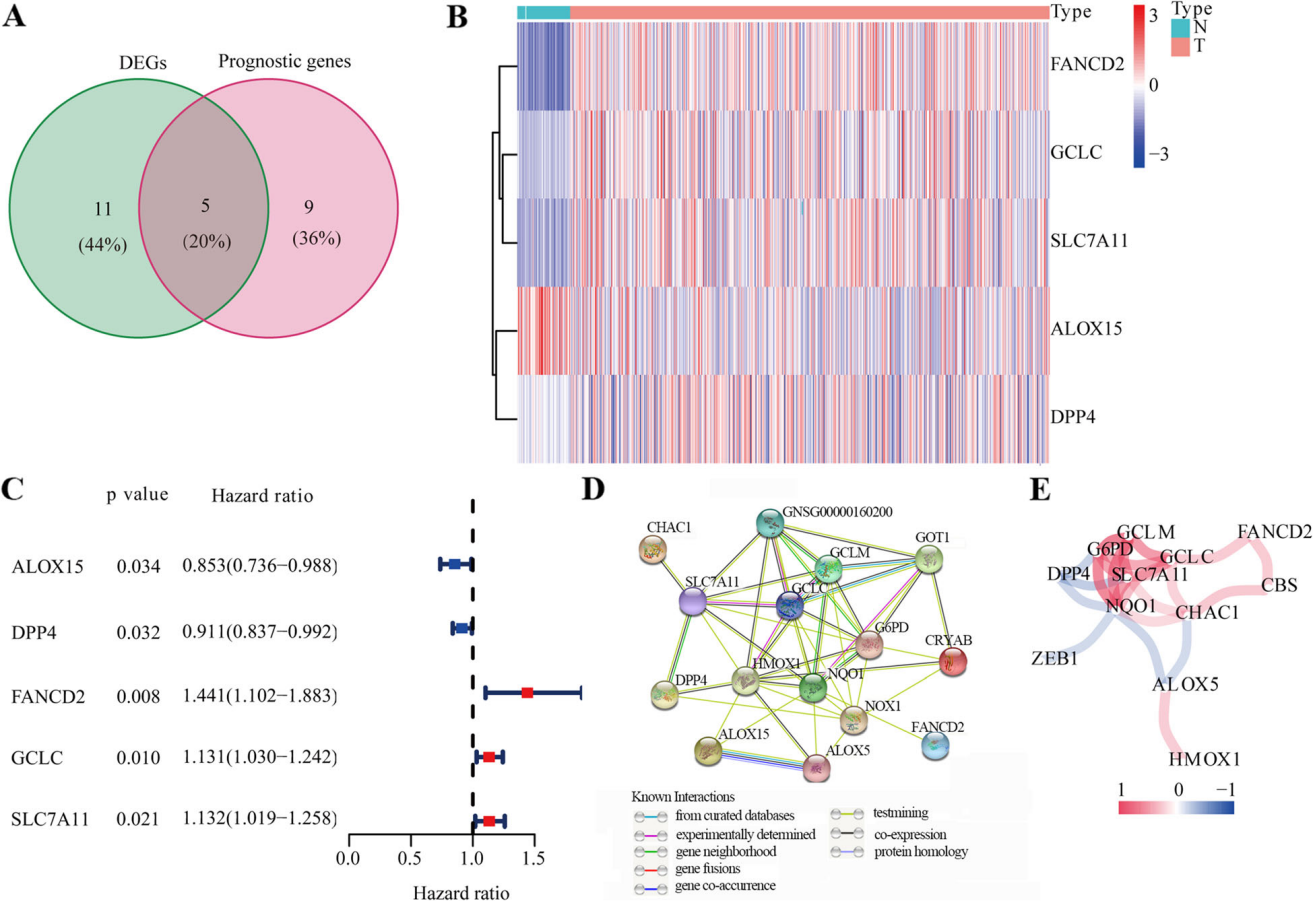
Identification of ferroptosis-related genes in TCGA cohort. (**a**) A Venn diagram of DEGs associated with OS. (**b**) Heatmap of five hub genes. (**c**) Univariate Cox regression analysis of five hub genes. (**d**) Protein–protein interaction network of ferroptosis-related genes. (**e**) Correlation network of ferroptosis-related genes

As shown in Fig. 1, NSCLC from TCGA–LUAD (*n* = 594) and the GEO (GSE13213 and GSE72904, *n* = 559) dataset were collected and analyzed. The detailed clinical characteristics of these patients are summarized in Table S2. Overall, we identified 16 DEGs (26.7%) between tumor and adjacent normal tissues and 14 (23.3%) prognostic genes in tumor samples (Fig. 2a). Univariate Cox regression analysis revealed that five of them—*ALOX5*, *DPP4*, *FANCD2*, *GCLC*, and *SLC7A11*—were both differentially expressed and correlated with OS (Fig. 2b-c). Interaction network analysis showed that *SLC7A11*, *GCLC*, *HMOX1*, *GCLM*, *G6PD*, *NQO1*, and *NOX1* were the significant hub genes (Fig. 2d-e), suggesting that they are mainly responsible for regulating ferroptosis in NSCLC.

### Development of a risk model in TCGA cohort

**Fig. 3:**
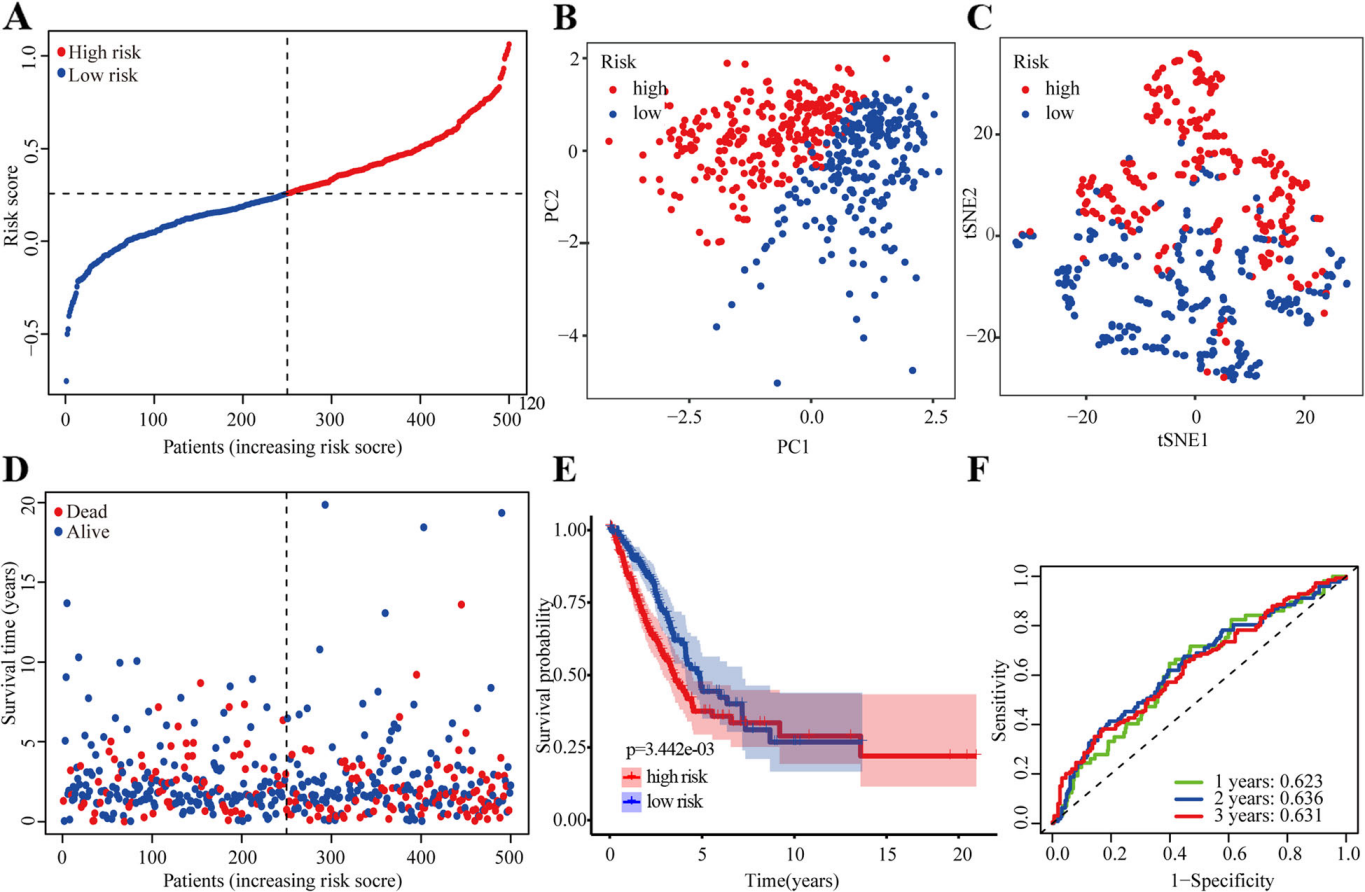
Prognostic analysis of the five-gene signature in TCGA cohort. (**a**) The risk scores of NSCLC in TCGA cohort. (**b**) PCA plot of NSCLC. (**c**) t-SNE analysis of NSCLC. (**d**) Distribution of survival status. (**e**) Survival analysis in the two risk subgroups. (**f**) AUC of the risk model

To develop a ferroptosis-related risk model, LASSO regression analysis was performed to construct a risk model based on the hub genes (*ALOX5*, *DPP4*, *FANCD2*, *GCLC*, and *SLC7A11*). High- (*n* = 297) and low-risk (*n =* 297) risk subgroups using the median cut-off value were found (Fig. 3a). PCA and t-SNE results also showed that our risk model could effectively stratify patients (Fig. 3b-c). Moreover, patients in the high-risk group showed a higher probability of earlier death than those in the low-risk group (Fig. 3d). The Kaplan–Meier OS curves revealed that patients in the high-risk group had a significantly worse OS than those in the low-risk group (Fig. 3e). Univariate [hazard ratio (HR): 2.97; 95% CI: 1.74–5.06; *P* < 0.001] and multivariate Cox regression analyses (HR: 2.70; 95% CI: 1.57–4.64; *P* < 0.001) also revealed that high-risk patients had a significantly worse OS than low-risk patients (Table S3). ROC analysis indicated that the area under the curve (AUC) for our model reached 0.792 at 1 year, 0.644 at 2 years, and 0.641 at 3 years (Fig. 3f). To evaluate the value of our ferroptosis-related risk model in indicating patient survival within the same clinical factor subgroup, patients were further stratified based on clinical parameters, such as age (≤65/> 65), gender (female/male), T stage (T1–2/T3–4), N stage (N0–1/N2–3), M stage (M0/M1), and clinical stage (I-II/III-IV). We found that the risk model could categorize patients in the early stage, particularly T1–2, N0–1, M0, and I-II clinical stages, into high- and low-risk subgroups (Fig. S1). Collectively, these results suggested that the model, composed of five ferroptosis-related genes, had a strong prognostic power.

### Risk model validation

**Fig. 4:**
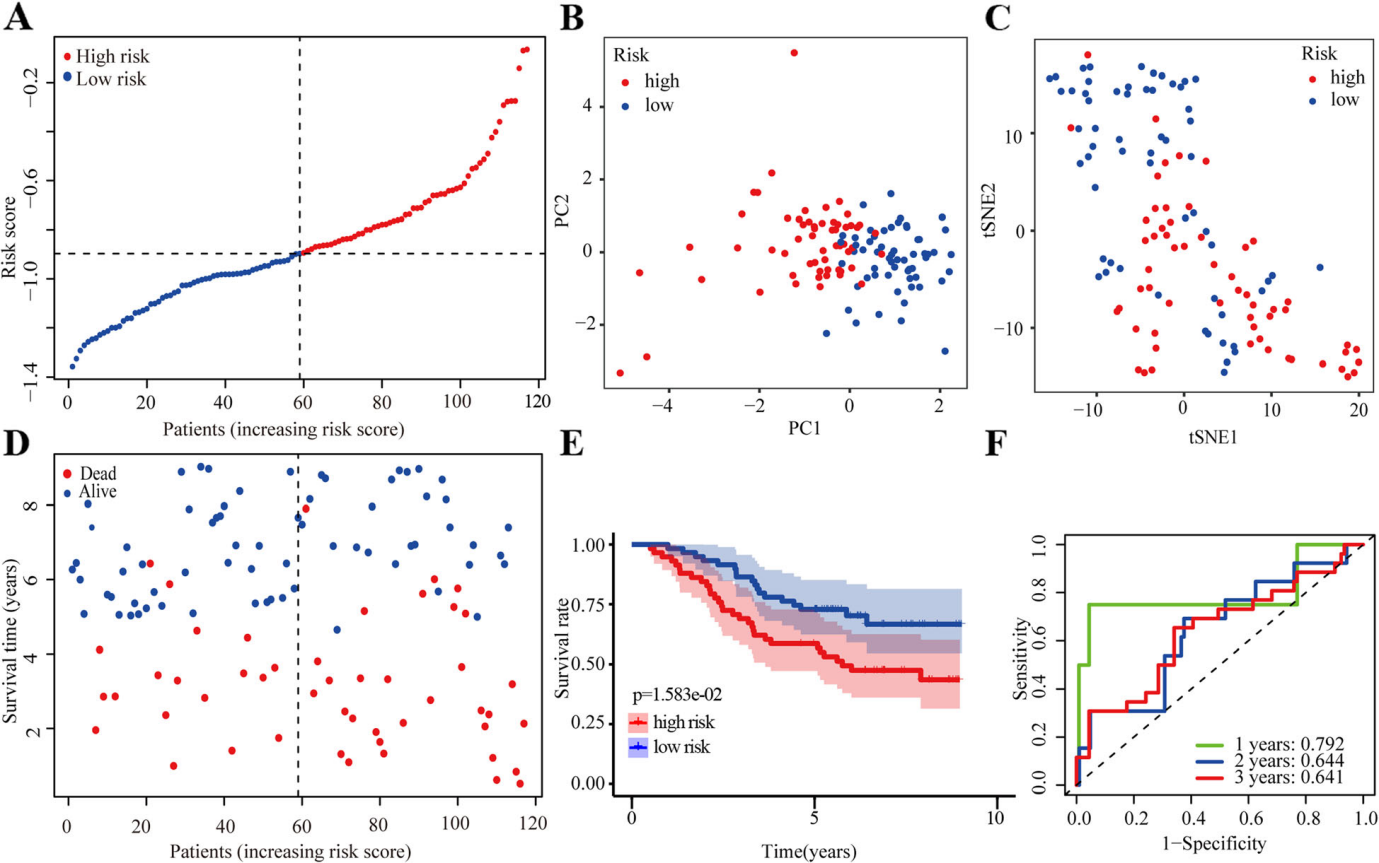
Validation of the risk model in the GSE13213 dataset. (**a**) The risk scores in the GSE13213 dataset. (**b**) PCA plot of GSE13213 dataset. (**c**) t-SNE analysis of of GSE13213 dataset. (**d**) Distribution of survival status. (**e**) Survival analysis in the two risk subgroups. (**f**) AUC of the risk model

We validated our risk model in the GSE13213 dataset. In total, 107 patients were stratified into high- and low-risk subgroups using median risk score values (Fig. 4a). PCA and t-SNE analysis suggested that patients were properly classified into high- and low-risk subgroups (Fig. 4b-c). Moreover, patients in the high-risk group showed a higher probability of earlier death (Fig. 4d) and had significantly worse OS than those in the low-risk group (Fig. 4e). The predictive power of our risk model was satisfactory (1 year, 0.792; 2 years, 0.644; and 3 years, 0.641) (Fig. 4f). Furthermore, the risk score was identified to be an independent predictor of OS by both univariate (HR: 5.18; 95% CI: 1.8–14.92; *P* < 0.01) and multivariate Cox regression (HR: 5.59; 95% CI: 1.79–17.44; *P* < 0.001) analysis (Table S3). To further verify the stringency of our risk model, the GSE72904 dataset was employed for model validation. Patients in this dataset were classified as being at a high or low risk; further, the survival of high-risk patients was significantly poor than that of low-risk ones (*P* < 0.001; Fig. S2a). The predictive power was as follows: 1 year, 0.635; 2 years, 0.634; and 3 years, 0.666 (Fig. S2b). Altogether, these findings suggested that our risk model had a strong predictive power in clinical applications.

### Functional enrichment analysis

**Fig. 5:**
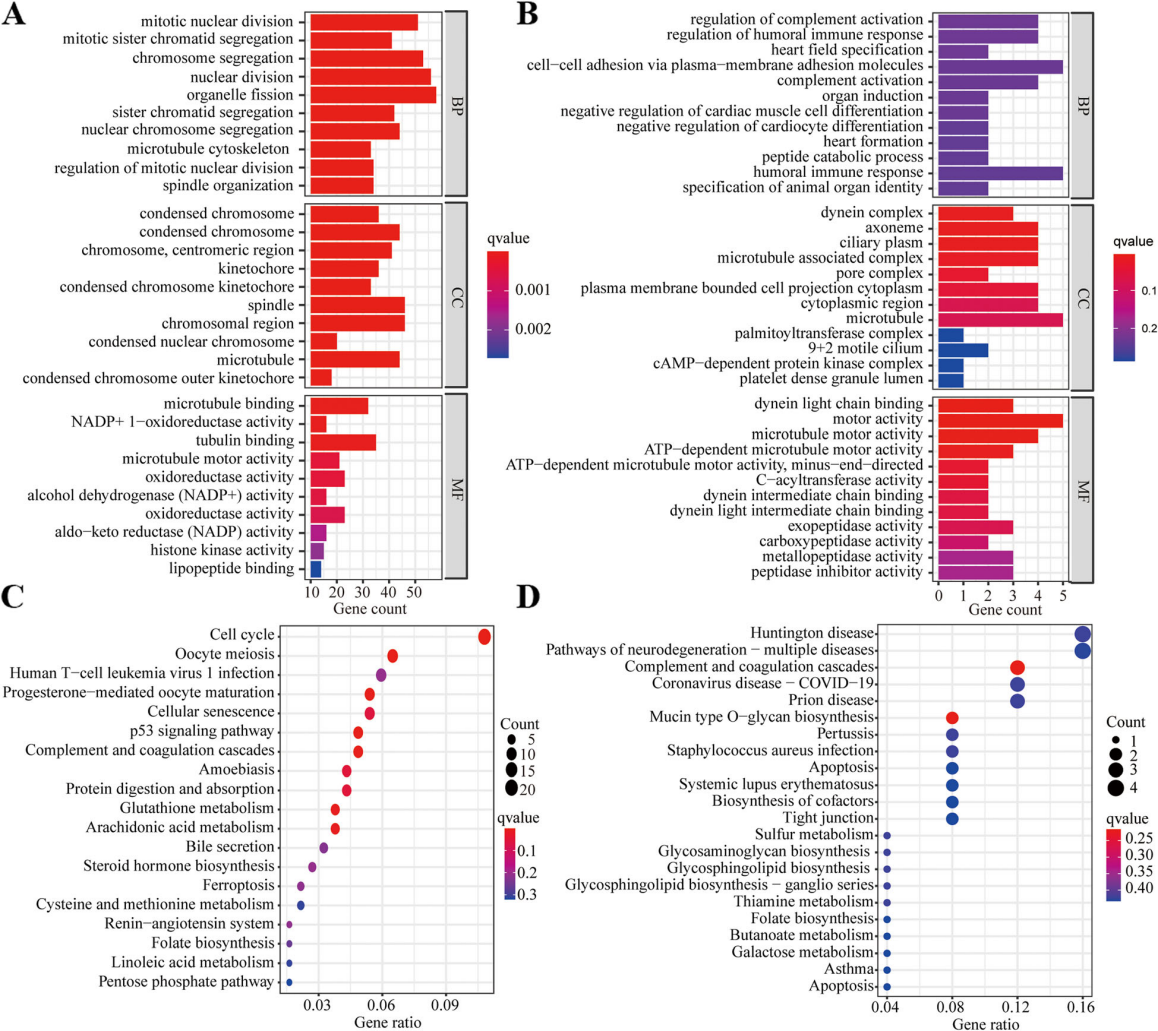
GO enrichment and KEGG pathway analysis. (**a-b**) GO enrichment analysis in TCGA cohort (**a**) and the GSE13213 dataset (**b**). (**c-d**) KEGG pathways analysis in TCGA cohort (**c**) and the GSE13213 dataset (**d**)

GO enrichment and KEGG pathway analyses were performed to explore the functional roles of DEGs in TGCA–LUAD cohort and the GSE13213 dataset. GO enrichment analysis showed that DEGs were mostly enriched in several immunity- and ferroptosis-related biological processes and molecular functions (*P* < 0.05; Fig. 5a-b). Further, KEGG pathway analysis showed that DEGs were mostly enriched in the ferroptosis pathway and immune-related pathways, such as human T-cell leukemia virus 1 (*HTLV-1*) infection pathway (*P* < 0.05; Fig. 5c-d). These findings suggested that there exists crosslinking between ferroptosis and tumor immunity in NSCLC.

**Fig. 6:**
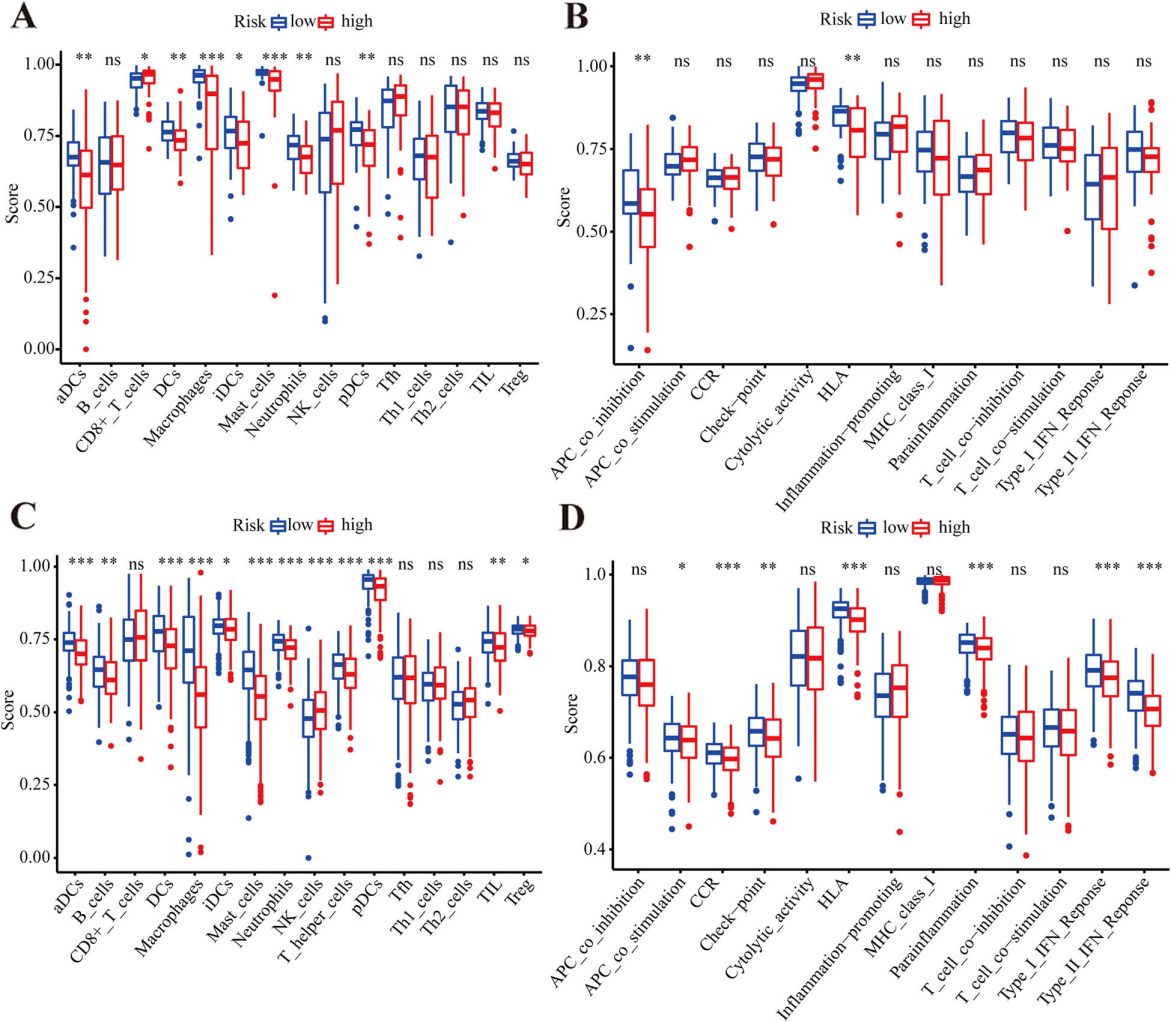
The ssGSEA scores between the risk subgroups. Immune scores and immune-related functions of different immune cells of TCGA cohort (**a-b**) and the GSE13213 dataset (**c-d**). CCR, cytokine–cytokine receptor. Adjusted *P* values are shown: ns, not significant; *, *P* < 0.05; **, *P* < 0.01; ***, *P* < 0.001

To further identify the immune status in different risk subgroups, ssGSEA was used to quantify the infiltrating scores of diverse immune cell subpopulations and immune-related functions/pathways. For immune cells, we found that the score of activated dendritic cells, immature dendritic cells, antigen-presenting cell co-stimulation, and human leukocyte antigen (HLA) was significantly different between the low- and high-risk subgroups in TCGA cohort (*P* < 0.05; Fig. 6a-b). The scores of antigen-presenting cell co-inhibition and HLA class were significantly lower in high-risk patients than in low-risk patients (*P* < 0.05; Fig. 6b). The GSE13213 dataset revealed differences in the scores of HLA class and type-I and -II immune interferon response (*P* < 0.05; Fig. 6c-d). More importantly, the immune score of the subgroups in both TCGA cohort and the GSE13213 dataset was significantly different, especially the score of macrophages and mast cells. These data were consistent with the findings of the functional enrichment analysis.

## Discussion

Cell death is an important aspect of mammalian development and homeostasis and is tightly integrated with the physiological and pathological state of an organism [20]. Ferroptosis is an iron-catalyzed form of regulated cell death, and iron accumulation and lipid peroxidation are the main biochemical characteristics of ferroptosis [21]. Recent studies have suggested that ferroptosis plays a key role in cancer development and treatment [22, 23]. However, immunity- and ferroptosis-related gene signatures remain largely uninvestigated in lung cancer. Herein we found that 44% ferroptosis-related genes were differentially expressed between lung tumor and adjacent normal tissues and that five ferroptosis-related genes were significantly associated with OS. A novel risk model was developed and validated in GEO dataset by five ferroptosis-related hub genes.

The risk model proposed in this study was composed of five ferroptosis-related genes: *FANCD2*, *GCLC*, *SLC7A11*, *ALOX15*, and *DPP4*. *FANCD2* is a nuclear protein involved in DNA damage repair and has been reported to protect against ferroptosis-mediated injury in cases of colon adenocarcinoma, clear cell renal cell carcinoma, and low-grade glioma [24–26]. Glutamate cysteine ligase is composed of the catalytic subunit *GCLC*, which evidently has a glutathione-independent, non-canonical role in conferring protection against ferroptosis, and this is achieved via the maintenance of glutamate homeostasis under cystine starvation [27–29]. The inhibition of *SLC7A11*-mediated cystine uptake can lead to intracellular glutathione deficiency, resulting in ferroptosis-mediated cell death [17, 21, 30]. *ALOX15* is closely associated to lipid ROS production in various types of tissues and tumors [31–33]. *DPP4*, a mitochondria-encoded gene, is responsible for ferroptosis induction [29].

In this study, functional analyses indicated that the identified DEGs were enriched in several immune-related pathways, including *HTLV-1* pathway, which has been implicated in many types of cancers [34–37]. HTLV-1 encodes two viral genes, namely Tax and *HTLV-1* bZIP factor (HBZ), which play a critical role in viral transcription and promotion of T-cell proliferation. HBZ, a suppressor of viral transcription, can change the immunophenotype of infected cells, conferring an effector regulatory T cell (eTreg)-like signature (*CD4*+ *CD25*+ *CCR4*+ *TIGIT*+ *Foxp*3+) and enhancing the proliferation of this subset [38–40]. We speculate that ferroptosis affects prognosis via the *HTLV-1* pathway, which drives the differentiation of Treg. Our findings also indicated that in comparison with low-risk patients, tumor-specific cellular immunity was altered in high-risk patients. Further, the ssGSEA score of HLA class was significantly lower in the high-risk group, indicating the immune suppression in the high-risk patients.

This study had several limitations. First, the retrospective data from public databases were used to construct and validate our risk model. Future prospective studies are needed to verify its clinical application. Second, the risk model was only associated with ferroptosis-related genes, the mutation status of oncogenic drivers, such as *EGFR* and *ALK*, were not included into the risk model, thus the model needs to be further improved. Last, our results relevant to the signature-based risk model and immune activity are theoretical and the potential mechanism needs to be further explored.

## Conclusions

We herein constructed a novel immunity- and ferroptosis-related risk model that can serve as a potential gene signature and therapeutic target for NSCLC. Although further studies are warranted to elucidate the mechanisms underlying tumor immunity, we believe that targeting both immunity- and ferroptosis-related genes should prove effective for treating lung cancer.

## Supplementary Information

**Additional file 1: Fig. S1.**. Stratified survival analyses and nomogram of the risk model (**a–f**) Kaplan–Meier survival curves for subgroups stratified by different clinical characteristics. Age ≤ 65/> 65 years (**a**), female/male (**b**), T1–2/3–4 (**c**), N0–1/2–3 (**d**), M0/1 (**e**), and clinical stage I-II/III-IV (**f**).

**Additional file 2: Fig. S2.** Validation of the five-gene signature in the GSE72904 dataset. (**a**) OS analysis of patients in the high- and low-risk subgroups. (**b**) AUC of the five-gene signature.

**Additional file 3: Table S1.** Ferroptosis-related genes.

**Additional file 4: Table S2.** Clinical characteristics of patients in TCGA cohort and the GSE13213 dataset.

**Additional file 5: Table S3.** Results of univariate and multivariate Cox regression analysis.

## Data Availability

The datasets used and/or analysed during the current study available from the corresponding author on reasonable request.

## Abbreviations

NSCLC: Non-small cell lung cancer
ROC: Receiver operating characteristic
OS: Overall survival
TCGA: The Cancer Genome Atlas
DEGs: Differentially expressed genes
GO: Gene ontology
KEGG: Kyoto Encyclopedia of Genes and Genomes
PCA: Principal Components Analysis
t-SNE: t-Distributed Stochastic Neighbor Embedding
ssGSEA: Single-sample gene set enrichment analysis
*HTLV-1*: Human T-cell leukemia virus 1
HLA: Human leukocyte antigen
