# Supplementary figures and images for: Molecular identification of an immunity- and Ferroptosis-related gene signature in non-small cell lung Cancer
Source: BMC Cancer. 2021 Jul 6;21:783. doi: 10.1186/s12885-021-08541-w (PMC8259362; doi:10.1186/s12885-021-08541-w)

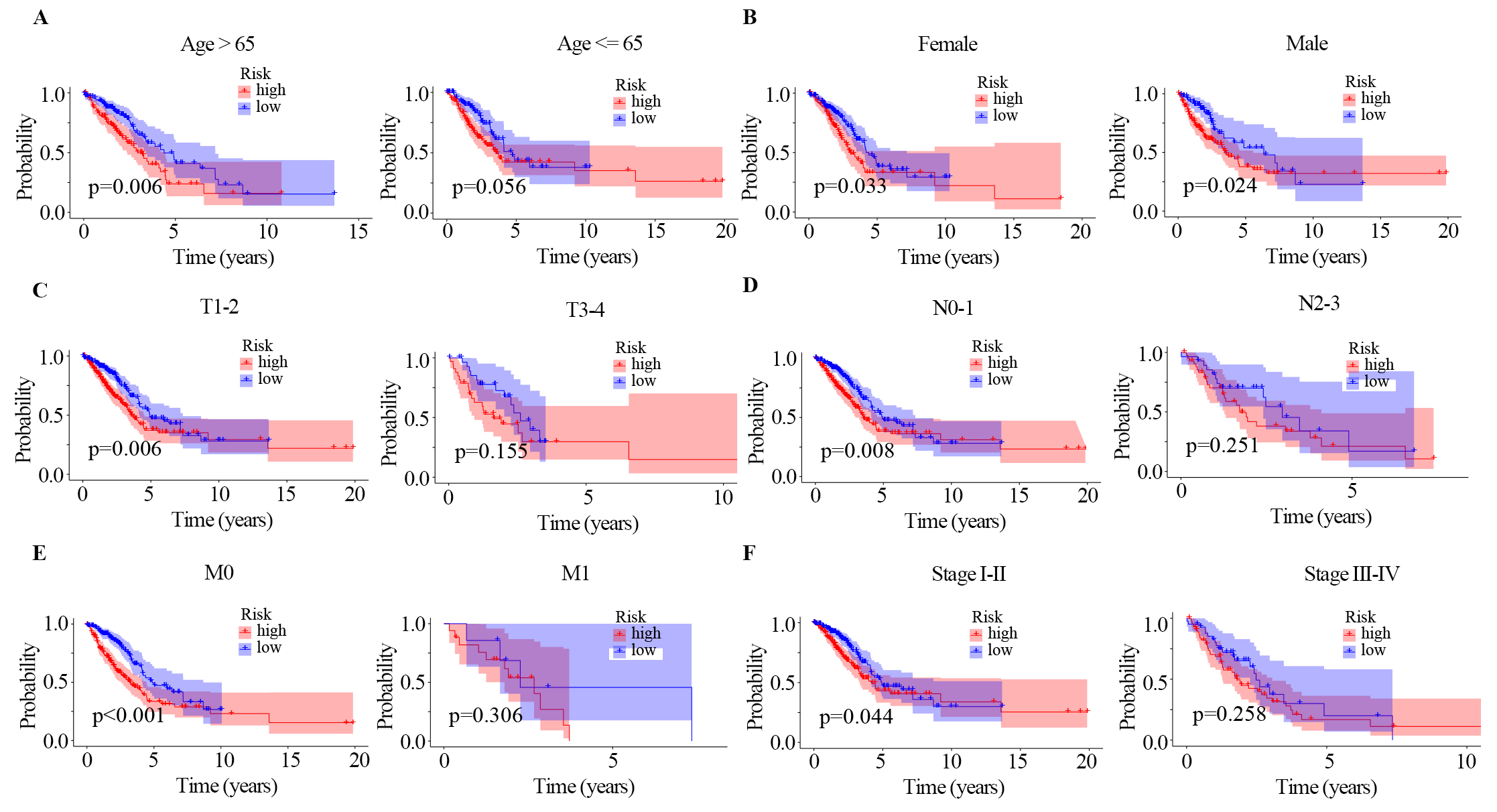

Supplement: Supplementary file 1 — Additional file 1: Fig. S1.. Stratified survival analyses and nomogram of the risk model (a–f) Kaplan–Meier survival curves for subgroups stratified by different clinical characteristics. Age ≤ 65/> 65 years (a), female/male (b), T1–2/3–4 (c), N0–1/2–3 (d), M0/1 (e), and clinical stage I-II/III-IV (f). [file 12885_2021_8541_MOESM1_ESM.tif]

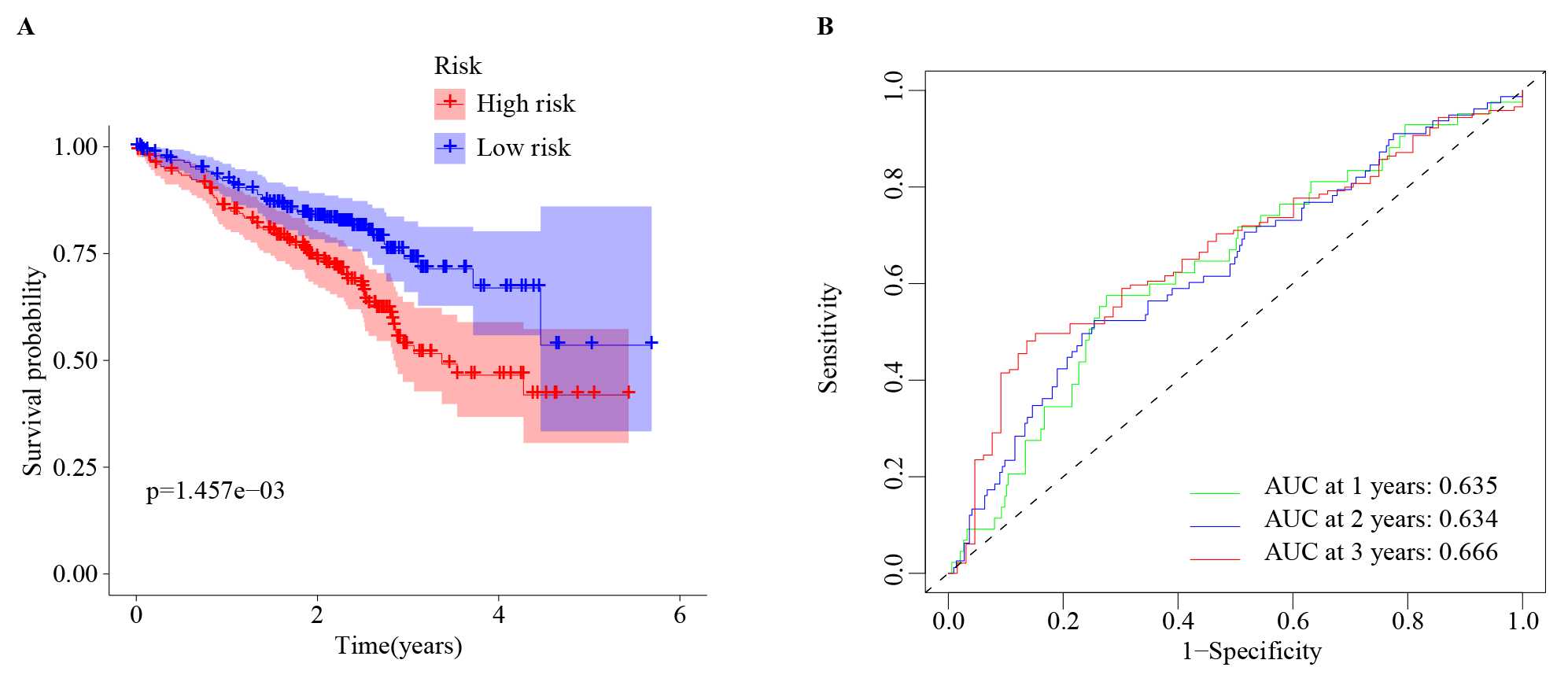

Supplement: Supplementary file 2 — Additional file 2: Fig. S2. Validation of the five-gene signature in the GSE72904 dataset. (a) OS analysis of patients in the high- and low-risk subgroups. (b) AUC of the five-gene signature. [file 12885_2021_8541_MOESM2_ESM.tif]
