# Supplementary material for: Molecular identification of an immunity- and Ferroptosis-related gene signature in non-small cell lung Cancer
Source: BMC Cancer. 2021 Jul 6;21:783. doi: 10.1186/s12885-021-08541-w (PMC8259362; doi:10.1186/s12885-021-08541-w)
Supplement: Supplementary file 4 — Additional file 4: Table S2. Clinical characteristics of patients in TCGA cohort and the GSE13213 dataset. [file 12885_2021_8541_MOESM4_ESM.docx]

| **Table S2**. Clinical characteristics of patients in the Cancer Genome Atlas Lung adenocarcinoma cohort and GSE13213 dataset. | | |
| --- | --- | --- |
| **Characteristics** | **TCGA LUAD** | **GSE13213** |
| **n** | 509 | 117 |
| **Age**  <=65  >65  Unknow | 240  259  10 | 78  39  - |
| **Gender (%)**  Female  Male | 274  235 | 57  60 |
| **Stage**  I  II  III  IV  Unknow | 273  122  81  25  8 | 79  13  25  -  - |
| **T**  T1  T2  T3  T4  Unknow | 171  272  45  18  3 | 54  50  8  5  - |
| **N**  N0  N1  N2  N3  Unknow | 329  97  70  2  11 | 87  8  44  -  - |
| **M**  M0  M1  Unknow | 341  24  144 | 117  -  - |
| LUAD: Lung adenocarcinoma; TCGA: The Cancer Genome Atlas; TNM: Tumor node metastasis. | | |
