# Supplementary material for: Molecular identification of an immunity- and Ferroptosis-related gene signature in non-small cell lung Cancer
Source: BMC Cancer. 2021 Jul 6;21:783. doi: 10.1186/s12885-021-08541-w (PMC8259362; doi:10.1186/s12885-021-08541-w)
Supplement: Supplementary file 5 — Additional file 5: Table S3. Results of univariate and multivariate Cox regression analysis. [file 12885_2021_8541_MOESM5_ESM.docx]

| **Table S3**. Results of univariate and multivariate Cox regression analyses. | | | | | | | | | | | | | | | |
| --- | --- | --- | --- | --- | --- | --- | --- | --- | --- | --- | --- | --- | --- | --- | --- |
|  | **TCGA LUAD** | | | | | | |  | **GSE13213** | | | | | | |
| Factors | Univariate | | |  | Multivariate | | |  | Univariate | | |  | Multivariate | | |
|  | HR | 95% CI | *P* value |  | HR | 95% CI | *P* value |  | HR | 95% CI | *P* value |  | HR | 95% CI | *P* value |
| Age | 1.19 | 0.89-1.61  0.81-1.46  1.80-3.37  1.74-5.06 | 0.2428 |  |  | 1.71-3.20  1.57-4.64 |  |  | 1.39 | 0.78-2.48  0.77-2.39  1.66-5.33  1.80-14.92 | 0.2576 |  |  | 1.63-5.25  1.79-17.44 |  |
| Gender | 1.09 |  | 0.5709 |  |  |  |  |  | 1.36 |  | 0.2858 |  |  |  |  |
| Tumor Stage | 2.47 |  | <0.0001 |  | 2.34 |  | <0.0001 |  | 2.97 |  | 0.0003 |  | 2.92 |  | 0.0003 |
| Risk Score | 2.97 |  | <0.0001 |  | 2.70 |  | 0.0003 |  | 5.18 |  | 0.0023 |  | 5.59 |  | 0.0030 |
| HR: Hazard ratio; LUAD: Lung adenocarcinoma; TCGA: The Cancer Genome Atlas. | | | | | | | | | | | | | | | |
